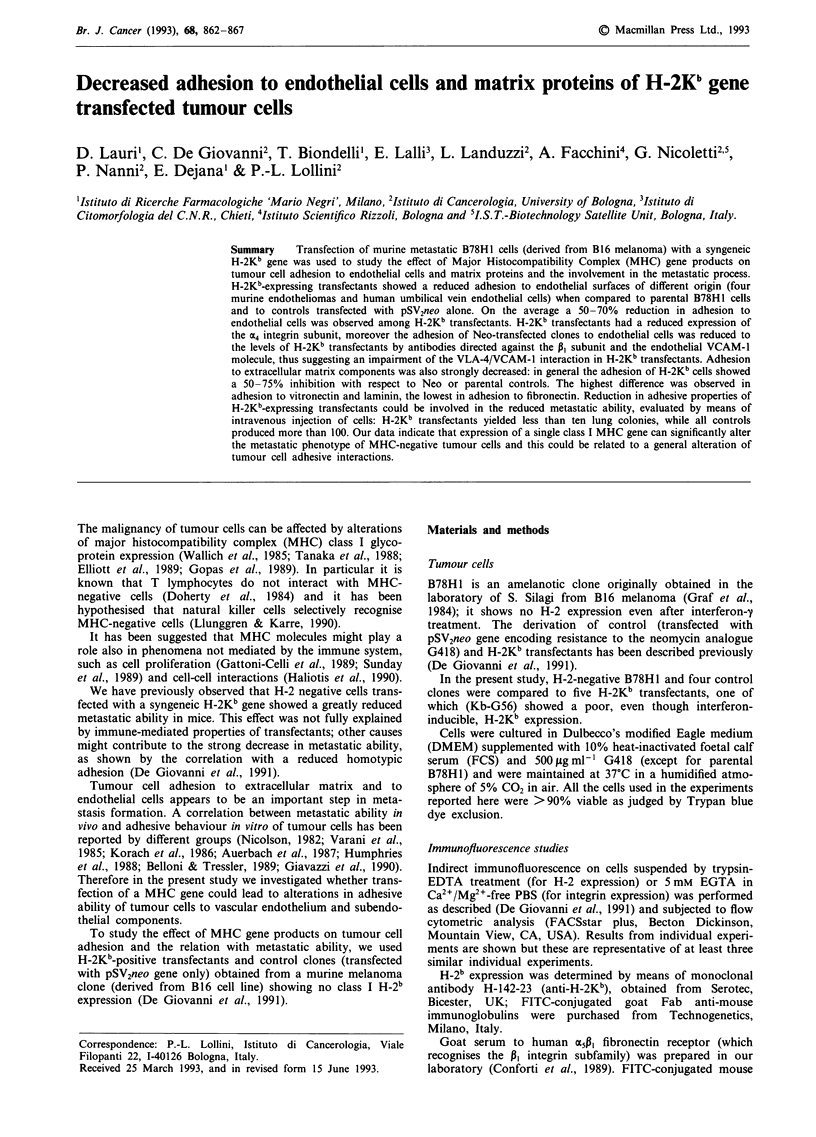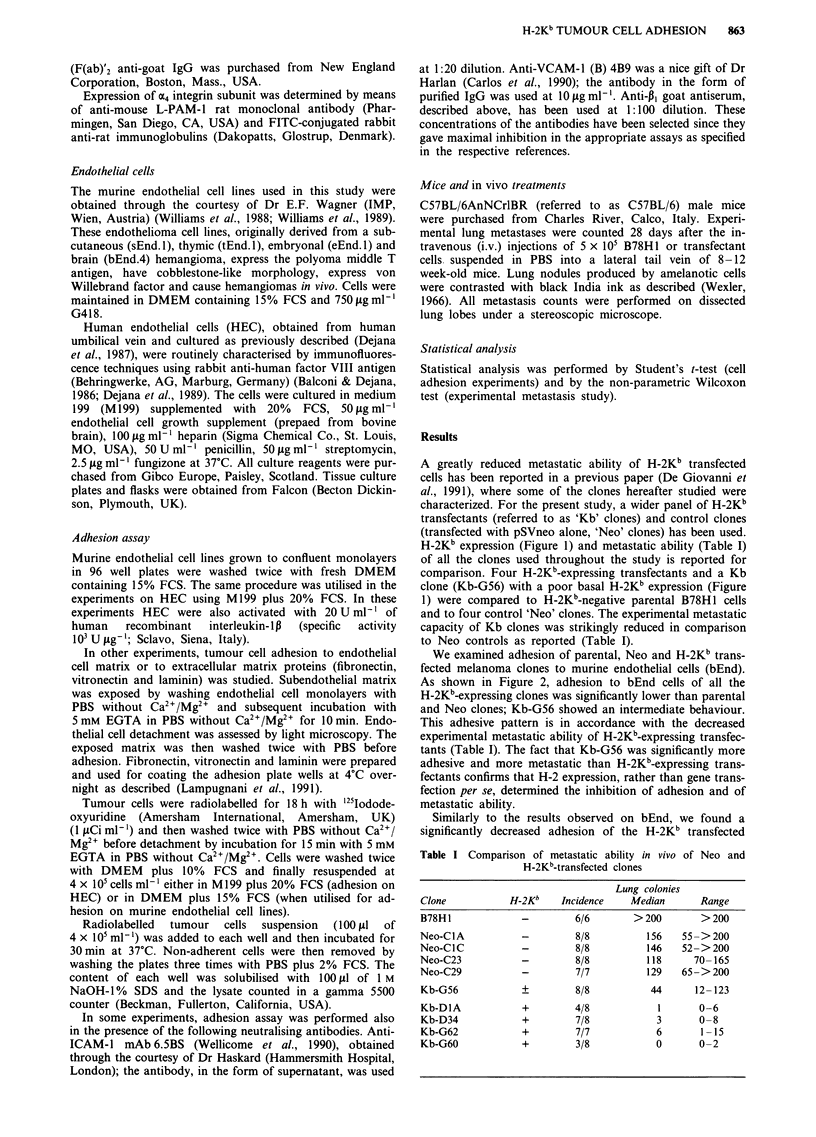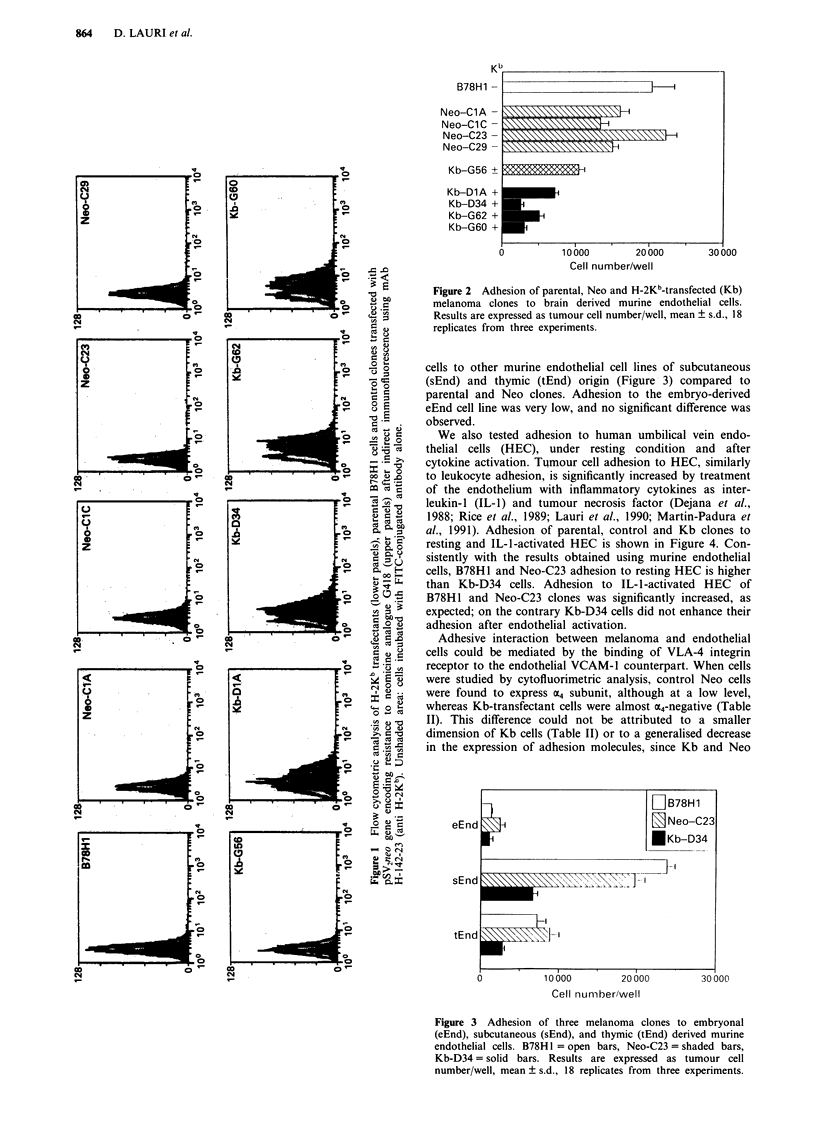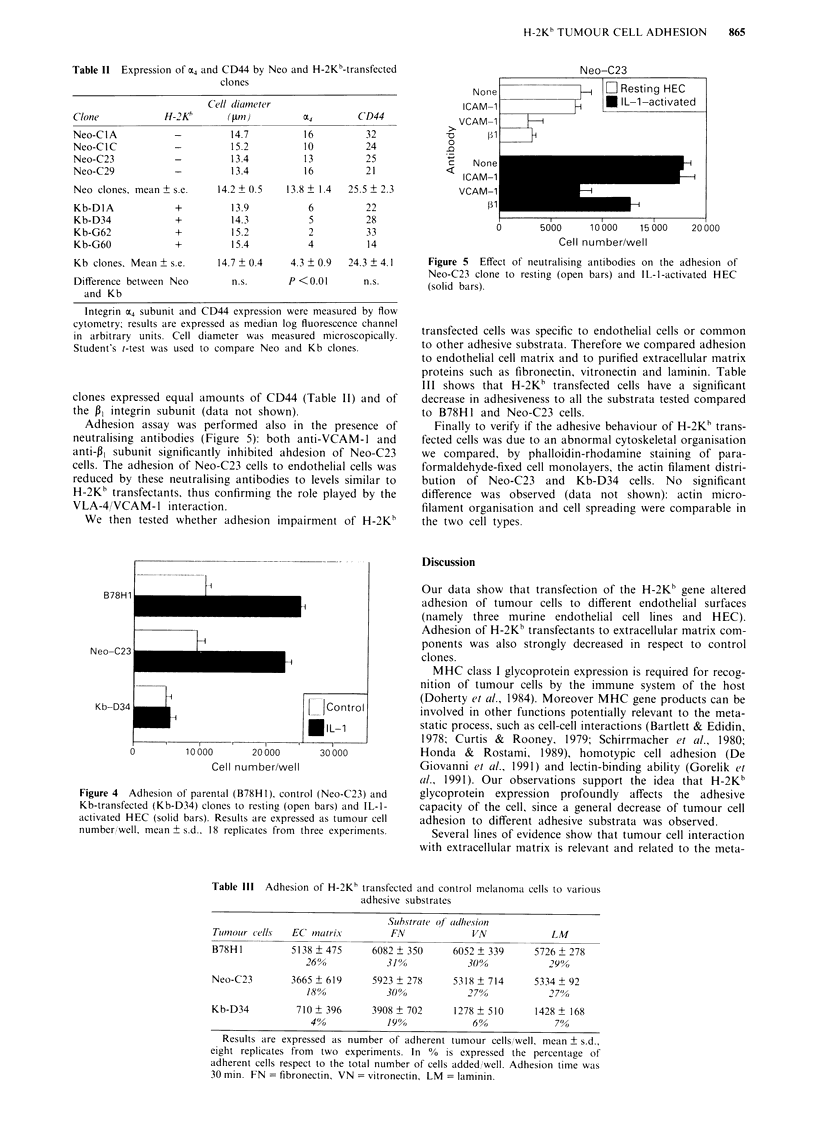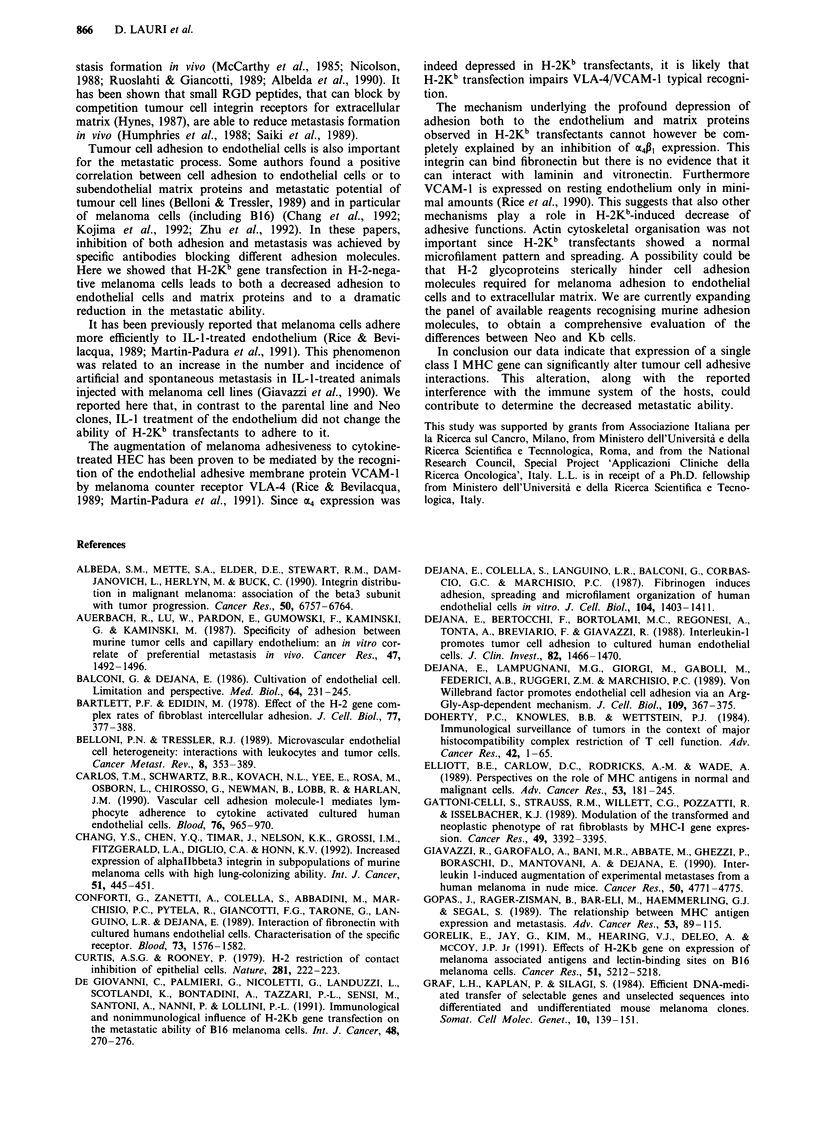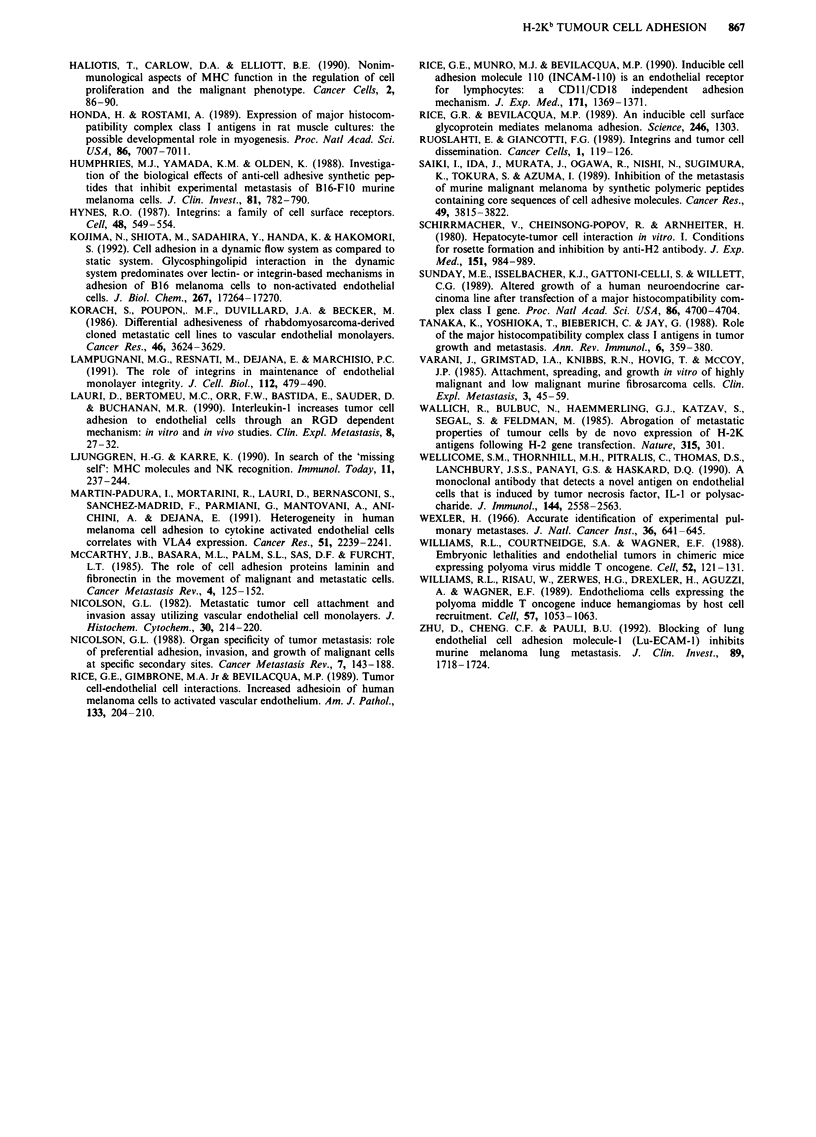# Decreased adhesion to endothelial cells and matrix proteins of H-2Kb gene transfected tumour cells.

**DOI:** 10.1038/bjc.1993.446

**Published:** 1993-11

**Authors:** D. Lauri, C. De Giovanni, T. Biondelli, E. Lalli, L. Landuzzi, A. Facchini, G. Nicoletti, P. Nanni, E. Dejana, P. L. Lollini

**Affiliations:** Istituto di Ricerche Farmacologiche Mario Negri, Milano, Italy.

## Abstract

Transfection of murine metastatic B78H1 cells (derived from B16 melanoma) with a syngeneic H-2Kb gene was used to study the effect of Major Histocompatibility Complex (MHC) gene products on tumour cell adhesion to endothelial cells and matrix proteins and the involvement in the metastatic process. H-2Kb-expressing transfectants showed a reduced adhesion to endothelial surfaces of different origin (four murine endotheliomas and human umbilical vein endothelial cells) when compared to parental B78H1 cells and to controls transfected with pSV2neo alone. On the average a 50-70% reduction in adhesion to endothelial cells was observed among H-2Kb transfectants. H-2Kb transfectants had a reduced expression of the alpha 4 integrin subunit, moreover the adhesion of Neo-transfected clones to endothelial cells was reduced to the levels of H-2Kb transfectants by antibodies directed against the beta 1 subunit and the endothelial VCAM-1 molecule, thus suggesting an impairment of the VLA-4/VCAM-1 interaction in H-2Kb transfectants. Adhesion to extracellular matrix components was also strongly decreased: in general the adhesion of H-2Kb cells showed a 50-75% inhibition with respect to Neo or parental controls. The highest difference was observed in adhesion to vitronectin and laminin, the lowest in adhesion to fibronectin. Reduction in adhesive properties of H-2Kb-expressing transfectants could be involved in the reduced metastatic ability, evaluated by means of intravenous injection of cells: H-2Kb transfectants yielded less than ten lung colonies, while all controls produced more than 100. Our data indicate that expression of a single class I MHC gene can significantly alter the metastatic phenotype of MHC-negative tumour cells and this could be related to a general alteration of tumour cell adhesive interactions.


Br. J. Cancer (1993), 68, 862–867 © Macmillan Press Ltd., 1993

### Decreased adhesion to endothelial cells and matrix proteins of H-2K$^b$ gene transfected tumour cells

D. Lauri[1], C. De Giovanni[2], T. Biondelli[1], E. Lalli[3], L. Landuzzi[2], A. Facchini[4], G. Nicoletti[2,5], P. Nanni[2], E. Dejana[1] & P.-L. Lollini[2]

[1]*Istituto di Ricerche Farmacologiche 'Mario Negri', Milano,* [2]*Istituto di Cancerologia, University of Bologna,* [3]*Istituto di Citomorfologia del C.N.R., Chieti,* [4]*Istituto Scientifico Rizzoli, Bologna and* [5]*I.S.T.-Biotechnology Satellite Unit, Bologna, Italy.*

**Summary** Transfection of murine metastatic B78H1 cells (derived from B16 melanoma) with a syngeneic H-2K$^b$ gene was used to study the effect of Major Histocompatibility Complex (MHC) gene products on tumour cell adhesion to endothelial cells and matrix proteins and the involvement in the metastatic process. H-2K$^b$-expressing transfectants showed a reduced adhesion to endothelial surfaces of different origin (four murine endotheliomas and human umbilical vein endothelial cells) when compared to parental B78H1 cells and to controls transfected with pSV$_2$*neo* alone. On the average a 50–70% reduction in adhesion to endothelial cells was observed among H-2K$^b$ transfectants. H-2K$^b$ transfectants had a reduced expression of the $\alpha_4$ integrin subunit, moreover the adhesion of Neo-transfected clones to endothelial cells was reduced to the levels of H-2K$^b$ transfectants by antibodies directed against the $\beta_1$ subunit and the endothelial VCAM-1 molecule, thus suggesting an impairment of the VLA-4/VCAM-1 interaction in H-2K$^b$ transfectants. Adhesion to extracellular matrix components was also strongly decreased: in general the adhesion of H-2K$^b$ cells showed a 50–75% inhibition with respect to Neo or parental controls. The highest difference was observed in adhesion to vitronectin and laminin, the lowest in adhesion to fibronectin. Reduction in adhesive properties of H-2K$^b$-expressing transfectants could be involved in the reduced metastatic ability, evaluated by means of intravenous injection of cells: H-2K$^b$ transfectants yielded less than ten lung colonies, while all controls produced more than 100. Our data indicate that expression of a single class I MHC gene can significantly alter the metastatic phenotype of MHC-negative tumour cells and this could be related to a general alteration of tumour cell adhesive interactions.

The malignancy of tumour cells can be affected by alterations of major histocompatibility complex (MHC) class I glycoprotein expression (Wallich *et al.*, 1985; Tanaka *et al.*, 1988; Elliott *et al.*, 1989; Gopas *et al.*, 1989). In particular it is known that T lymphocytes do not interact with MHC-negative cells (Doherty *et al.*, 1984) and it has been hypothesised that natural killer cells selectively recognise MHC-negative cells (Llunggren & Karre, 1990).

It has been suggested that MHC molecules might play a role also in phenomena not mediated by the immune system, such as cell proliferation (Gattoni-Celli *et al.*, 1989; Sunday *et al.*, 1989) and cell-cell interactions (Haliotis *et al.*, 1990).

We have previously observed that H-2 negative cells transfected with a syngeneic H-2K$^b$ gene showed a greatly reduced metastatic ability in mice. This effect was not fully explained by immune-mediated properties of transfectants; other causes might contribute to the strong decrease in metastatic ability, as shown by the correlation with a reduced homotypic adhesion (De Giovanni *et al.*, 1991).

Tumour cell adhesion to extracellular matrix and to endothelial cells appears to be an important step in metastasis formation. A correlation between metastatic ability *in vivo* and adhesive behaviour *in vitro* of tumour cells has been reported by different groups (Nicolson, 1982; Varani *et al.*, 1985; Korach *et al.*, 1986; Auerbach *et al.*, 1987; Humphries *et al.*, 1988; Belloni & Tressler, 1989; Giavazzi *et al.*, 1990). Therefore in the present study we investigated whether transfection of a MHC gene could lead to alterations in adhesive ability of tumour cells to vascular endothelium and subendothelial components.

To study the effect of MHC gene products on tumour cell adhesion and the relation with metastatic ability, we used H-2K$^b$-positive transfectants and control clones (transfected with pSV$_2$*neo* gene only) obtained from a murine melanoma clone (derived from B16 cell line) showing no class I H-2$^b$ expression (De Giovanni *et al.*, 1991).

Correspondence: P.-L. Lollini, Istituto di Cancerologia, Viale Filopanti 22, I-40126 Bologna, Italy.
Received 25 March 1993, and in revised form 15 June 1993.

### Materials and methods

### *Tumour cells*

B78H1 is an amelanotic clone originally obtained in the laboratory of S. Silagi from B16 melanoma (Graf *et al.*, 1984); it shows no H-2 expression even after interferon-$\gamma$ treatment. The derivation of control (transfected with pSV$_2$*neo* gene encoding resistance to the neomycin analogue G418) and H-2K$^b$ transfectants has been described previously (De Giovanni *et al.*, 1991).

In the present study, H-2-negative B78H1 and four control clones were compared to five H-2K$^b$ transfectants, one of which (Kb-G56) showed a poor, even though interferon-inducible, H-2K$^b$ expression.

Cells were cultured in Dulbecco's modified Eagle medium (DMEM) supplemented with 10% heat-inactivated foetal calf serum (FCS) and 500 $\mu g\,ml^{-1}$ G418 (except for parental B78H1) and were maintained at 37°C in a humidified atmosphere of 5% $CO_2$ in air. All the cells used in the experiments reported here were >90% viable as judged by Trypan blue dye exclusion.

### *Immunofluorescence studies*

Indirect immunofluorescence on cells suspended by trypsin-EDTA treatment (for H-2 expression) or 5 mM EGTA in $Ca^{2+}/Mg^{2+}$-free PBS (for integrin expression) was performed as described (De Giovanni *et al.*, 1991) and subjected to flow cytometric analysis (FACSstar plus, Becton Dickinson, Mountain View, CA, USA). Results from individual experiments are shown but these are representative of at least three similar individual experiments.

H-2$^b$ expression was determined by means of monoclonal antibody H-142-23 (anti-H-2K$^b$), obtained from Serotec, Bicester, UK; FITC-conjugated goat Fab anti-mouse immunoglobulins were purchased from Technogenetics, Milano, Italy.

Goat serum to human $\alpha_5\beta_1$ fibronectin receptor (which recognises the $\beta_1$ integrin subfamily) was prepared in our laboratory (Conforti *et al.*, 1989). FITC-conjugated mouse

(F(ab)'$_2$ anti-goat IgG was purchased from New England Corporation, Boston, Mass., USA.

Expression of $\alpha_4$ integrin subunit was determined by means of anti-mouse L-PAM-1 rat monoclonal antibody (Pharmingen, San Diego, CA, USA) and FITC-conjugated rabbit anti-rat immunoglobulins (Dakopatts, Glostrup, Denmark).

*Endothelial cells*

The murine endothelial cell lines used in this study were obtained through the courtesy of Dr E.F. Wagner (IMP, Wien, Austria) (Williams *et al.*, 1988; Williams *et al.*, 1989). These endothelioma cell lines, originally derived from a subcutaneous (sEnd.1), thymic (tEnd.1), embryonal (eEnd.1) and brain (bEnd.4) hemangioma, express the polyoma middle T antigen, have cobblestone-like morphology, express von Willebrand factor and cause hemangiomas *in vivo*. Cells were maintained in DMEM containing 15% FCS and 750 $\mu$g ml$^{-1}$ G418.

Human endothelial cells (HEC), obtained from human umbilical vein and cultured as previously described (Dejana *et al.*, 1987), were routinely characterised by immunofluorescence techniques using rabbit anti-human factor VIII antigen (Behringwerke, AG, Marburg, Germany) (Balconi & Dejana, 1986; Dejana *et al.*, 1989). The cells were cultured in medium 199 (M199) supplemented with 20% FCS, 50 $\mu$g ml$^{-1}$ endothelial cell growth supplement (prepaed from bovine brain), 100 $\mu$g ml$^{-1}$ heparin (Sigma Chemical Co., St. Louis, MO, USA), 50 U ml$^{-1}$ penicillin, 50 $\mu$g ml$^{-1}$ streptomycin, 2.5 $\mu$g ml$^{-1}$ fungizone at 37°C. All culture reagents were purchased from Gibco Europe, Paisley, Scotland. Tissue culture plates and flasks were obtained from Falcon (Becton Dickinson, Plymouth, UK).

*Adhesion assay*

Murine endothelial cell lines grown to confluent monolayers in 96 well plates were washed twice with fresh DMEM containing 15% FCS. The same procedure was utilised in the experiments on HEC using M199 plus 20% FCS. In these experiments HEC were also activated with 20 U ml$^{-1}$ of human recombinant interleukin-1$\beta$ (specific activity $10^3$ U $\mu$g$^{-1}$; Sclavo, Siena, Italy).

In other experiments, tumour cell adhesion to endothelial cell matrix or to extracellular matrix proteins (fibronectin, vitronectin and laminin) was studied. Subendothelial matrix was exposed by washing endothelial cell monolayers with PBS without $Ca^{2+}/Mg^{2+}$ and subsequent incubation with 5 mM EGTA in PBS without $Ca^{2+}/Mg^{2+}$ for 10 min. Endothelial cell detachment was assessed by light microscopy. The exposed matrix was then washed twice with PBS before adhesion. Fibronectin, vitronectin and laminin were prepared and used for coating the adhesion plate wells at 4°C overnight as described (Lampugnani *et al.*, 1991).

Tumour cells were radiolabelled for 18 h with $^{125}$Iododeoxyuridine (Amersham International, Amersham, UK) (1 $\mu$Ci ml$^{-1}$) and then washed twice with PBS without $Ca^{2+}/Mg^{2+}$ before detachment by incubation for 15 min with 5 mM EGTA in PBS without $Ca^{2+}/Mg^{2+}$. Cells were washed twice with DMEM plus 10% FCS and finally resuspended at $4 \times 10^5$ cells ml$^{-1}$ either in M199 plus 20% FCS (adhesion on HEC) or in DMEM plus 15% FCS (when utilised for adhesion on murine endothelial cell lines).

Radiolabelled tumour cells suspension (100 $\mu$l of $4 \times 10^5$ ml$^{-1}$) was added to each well and then incubated for 30 min at 37°C. Non-adherent cells were then removed by washing the plates three times with PBS plus 2% FCS. The content of each well was solubilised with 100 $\mu$l of 1 M NaOH-1% SDS and the lysate counted in a gamma 5500 counter (Beckman, Fullerton, California, USA).

In some experiments, adhesion assay was performed also in the presence of the following neutralising antibodies. Anti-ICAM-1 mAb 6.5BS (Wellicome *et al.*, 1990), obtained through the courtesy of Dr Haskard (Hammersmith Hospital, London); the antibody, in the form of supernatant, was used at 1:20 dilution. Anti-VCAM-1 (B) 4B9 was a nice gift of Dr Harlan (Carlos *et al.*, 1990); the antibody in the form of purified IgG was used at 10 $\mu$g ml$^{-1}$. Anti-$\beta_1$ goat antiserum, described above, has been used at 1:100 dilution. These concentrations of the antibodies have been selected since they gave maximal inhibition in the appropriate assays as specified in the respective references.

*Mice and* in vivo *treatments*

C57BL/6AnNCrlBR (referred to as C57BL/6) male mice were purchased from Charles River, Calco, Italy. Experimental lung metastases were counted 28 days after the intravenous (i.v.) injections of $5 \times 10^5$ B78H1 or transfectant cells suspended in PBS into a lateral tail vein of 8–12 week-old mice. Lung nodules produced by amelanotic cells were contrasted with black India ink as described (Wexler, 1966). All metastasis counts were performed on dissected lung lobes under a stereoscopic microscope.

*Statistical analysis*

Statistical analysis was performed by Student's *t*-test (cell adhesion experiments) and by the non-parametric Wilcoxon test (experimental metastasis study).

### Results

A greatly reduced metastatic ability of H-2K$^b$ transfected cells has been reported in a previous paper (De Giovanni *et al.*, 1991), where some of the clones hereafter studied were characterized. For the present study, a wider panel of H-2K$^b$ transfectants (referred to as 'Kb' clones) and control clones (transfected with pSVneo alone, 'Neo' clones) has been used. H-2K$^b$ expression (Figure 1) and metastatic ability (Table I) of all the clones used throughout the study is reported for comparison. Four H-2K$^b$-expressing transfectants and a Kb clone (Kb-G56) with a poor basal H-2K$^b$ expression (Figure 1) were compared to H-2K$^b$-negative parental B78H1 cells and to four control 'Neo' clones. The experimental metastatic capacity of Kb clones was strikingly reduced in comparison to Neo controls as reported (Table I).

We examined adhesion of parental, Neo and H-2K$^b$ transfected melanoma clones to murine endothelial cells (bEnd). As shown in Figure 2, adhesion to bEnd cells of all the H-2K$^b$-expressing clones was significantly lower than parental and Neo clones; Kb-G56 showed an intermediate behaviour. This adhesive pattern is in accordance with the decreased experimental metastatic ability of H-2K$^b$-expressing transfectants (Table I). The fact that Kb-G56 was significantly more adhesive and more metastatic than H-2K$^b$-expressing transfectants confirms that H-2 expression, rather than gene transfection *per se*, determined the inhibition of adhesion and of metastatic ability.

Similarly to the results observed on bEnd, we found a significantly decreased adhesion of the H-2K$^b$ transfected

**Table I** Comparison of metastatic ability *in vivo* of Neo and H-2K$^b$-transfected clones

| *Clone* | *H-2K$^b$* | *Incidence* | *Lung colonies Median* | *Range* |
|---|---|---|---|---|
| B78H1 | – | 6/6 | >200 | >200 |
| Neo-C1A | – | 8/8 | 156 | 55->200 |
| Neo-C1C | – | 8/8 | 146 | 52->200 |
| Neo-C23 | – | 8/8 | 118 | 70-165 |
| Neo-C29 | – | 7/7 | 129 | 65->200 |
| Kb-G56 | ± | 8/8 | 44 | 12-123 |
| Kb-D1A | + | 4/8 | 1 | 0-6 |
| Kb-D34 | + | 7/8 | 3 | 0-8 |
| Kb-G62 | + | 7/7 | 6 | 1-15 |
| Kb-G60 | + | 3/8 | 0 | 0-2 |

**Figure 1** Flow cytometric analysis of H-2K$^b$ transfectants (lower panels), parental B78H1 cells and control clones transfected with pSV$_2$*neo* gene encoding resistance to neomicine analogue G418 (upper panels) after indirect immunofluorescence using mAb H-142-23 (anti H-2K$^b$). Unshaded area: cells incubated with FITC-conjugated antibody alone.

**Figure 2** Adhesion of parental, Neo and H-2K$^b$-transfected (Kb) melanoma clones to brain derived murine endothelial cells. Results are expressed as tumour cell number/well, mean ± s.d., 18 replicates from three experiments.

cells to other murine endothelial cell lines of subcutaneous (sEnd) and thymic (tEnd) origin (Figure 3) compared to parental and Neo clones. Adhesion to the embryo-derived eEnd cell line was very low, and no significant difference was observed.

We also tested adhesion to human umbilical vein endothelial cells (HEC), under resting condition and after cytokine activation. Tumour cell adhesion to HEC, similarly to leukocyte adhesion, is significantly increased by treatment of the endothelium with inflammatory cytokines as interleukin-1 (IL-1) and tumour necrosis factor (Dejana *et al.*, 1988; Rice *et al.*, 1989; Lauri *et al.*, 1990; Martin-Padura *et al.*, 1991). Adhesion of parental, control and Kb clones to resting and IL-1-activated HEC is shown in Figure 4. Consistently with the results obtained using murine endothelial cells, B78H1 and Neo-C23 adhesion to resting HEC is higher than Kb-D34 cells. Adhesion to IL-1-activated HEC of B78H1 and Neo-C23 clones was significantly increased, as expected; on the contrary Kb-D34 cells did not enhance their adhesion after endothelial activation.

Adhesive interaction between melanoma and endothelial cells could be mediated by the binding of VLA-4 integrin receptor to the endothelial VCAM-1 counterpart. When cells were studied by cytofluorimetric analysis, control Neo cells were found to express $\alpha_4$ subunit, although at a low level, whereas Kb-transfectant cells were almost $\alpha_4$-negative (Table II). This difference could not be attributed to a smaller dimension of Kb cells (Table II) or to a generalised decrease in the expression of adhesion molecules, since Kb and Neo

**Figure 3** Adhesion of three melanoma clones to embryonal (eEnd), subcutaneous (sEnd), and thymic (tEnd) derived murine endothelial cells. B78H1 = open bars, Neo-C23 = shaded bars, Kb-D34 = solid bars. Results are expressed as tumour cell number/well, mean ± s.d., 18 replicates from three experiments.

**Table II** Expression of $\alpha_4$ and CD44 by Neo and H-2K$^b$-transfected clones

| *Clone* | *H-2K$^b$* | *Cell diameter (μm)* | *$\alpha_4$* | *CD44* |
|---|---|---|---|---|
| Neo-C1A | – | 14.7 | 16 | 32 |
| Neo-C1C | – | 15.2 | 10 | 24 |
| Neo-C23 | – | 13.4 | 13 | 25 |
| Neo-C29 | – | 13.4 | 16 | 21 |
| Neo clones, mean ± s.e. | | 14.2 ± 0.5 | 13.8 ± 1.4 | 25.5 ± 2.3 |
| Kb-D1A | + | 13.9 | 6 | 22 |
| Kb-D34 | + | 14.3 | 5 | 28 |
| Kb-G62 | + | 15.2 | 2 | 33 |
| Kb-G60 | + | 15.4 | 4 | 14 |
| Kb clones, Mean ± s.e. | | 14.7 ± 0.4 | 4.3 ± 0.9 | 24.3 ± 4.1 |
| Difference between Neo and Kb | | n.s. | $P<0.01$ | n.s. |

Integrin $\alpha_4$ subunit and CD44 expression were measured by flow cytometry; results are expressed as median log fluorescence channel in arbitrary units. Cell diameter was measured microscopically. Student's *t*-test was used to compare Neo and Kb clones.

clones expressed equal amounts of CD44 (Table II) and of the $\beta_1$ integrin subunit (data not shown).

Adhesion assay was performed also in the presence of neutralising antibodies (Figure 5): both anti-VCAM-1 and anti-$\beta_1$ subunit significantly inhibited ahdesion of Neo-C23 cells. The adhesion of Neo-C23 cells to endothelial cells was reduced by these neutralising antibodies to levels similar to H-2K$^b$ transfectants, thus confirming the role played by the VLA-4/VCAM-1 interaction.

We then tested whether adhesion impairment of H-2K$^b$ transfected cells was specific to endothelial cells or common to other adhesive substrata. Therefore we compared adhesion to endothelial cell matrix and to purified extracellular matrix proteins such as fibronectin, vitronectin and laminin. Table III shows that H-2K$^b$ transfected cells have a significant decrease in adhesiveness to all the substrata tested compared to B78H1 and Neo-C23 cells.

Finally to verify if the adhesive behaviour of H-2K$^b$ transfected cells was due to an abnormal cytoskeletal organisation we compared, by phalloidin-rhodamine staining of paraformaldehyde-fixed cell monolayers, the actin filament distribution of Neo-C23 and Kb-D34 cells. No significant difference was observed (data not shown): actin microfilament organisation and cell spreading were comparable in the two cell types.

**Figure 5** Effect of neutralising antibodies on the adhesion of Neo-C23 clone to resting (open bars) and IL-1-activated HEC (solid bars).

**Figure 4** Adhesion of parental (B78H1), control (Neo-C23) and Kb-transfected (Kb-D34) clones to resting (open bars) and IL-1-activated HEC (solid bars). Results are expressed as tumour cell number/well, mean ± s.d., 18 replicates from three experiments.

### Discussion

Our data show that transfection of the H-2K$^b$ gene altered adhesion of tumour cells to different endothelial surfaces (namely three murine endothelial cell lines and HEC). Adhesion of H-2K$^b$ transfectants to extracellular matrix components was also strongly decreased in respect to control clones.

MHC class I glycoprotein expression is required for recognition of tumour cells by the immune system of the host (Doherty *et al.*, 1984). Moreover MHC gene products can be involved in other functions potentially relevant to the metastatic process, such as cell-cell interactions (Bartlett & Edidin, 1978; Curtis & Rooney, 1979; Schirrmacher *et al.*, 1980; Honda & Rostami, 1989), homotypic cell adhesion (De Giovanni *et al.*, 1991) and lectin-binding ability (Gorelik *et al.*, 1991). Our observations support the idea that H-2K$^b$ glycoprotein expression profoundly affects the adhesive capacity of the cell, since a general decrease of tumour cell adhesion to different adhesive substrata was observed.

Several lines of evidence show that tumour cell interaction with extracellular matrix is relevant and related to the meta-

**Table III** Adhesion of H-2K$^b$ transfected and control melanoma cells to various adhesive substrates

| *Tumour cells* | *Substrate of adhesion* EC matrix | FN | VN | LM |
|---|---|---|---|---|
| B78H1 | 5138 ± 475<br>*26%* | 6082 ± 350<br>*31%* | 6052 ± 339<br>*30%* | 5726 ± 278<br>*29%* |
| Neo-C23 | 3665 ± 619<br>*18%* | 5923 ± 278<br>*30%* | 5318 ± 714<br>*27%* | 5334 ± 92<br>*27%* |
| Kb-D34 | 710 ± 396<br>*4%* | 3908 ± 702<br>*19%* | 1278 ± 510<br>*6%* | 1428 ± 168<br>*7%* |

Results are expressed as number of adherent tumour cells/well, mean ± s.d., eight replicates from two experiments. In % is expressed the percentage of adherent cells respect to the total number of cells added/well. Adhesion time was 30 min. FN = fibronectin, VN = vitronectin, LM = laminin.

stasis formation *in vivo* (McCarthy *et al.*, 1985; Nicolson, 1988; Ruoslahti & Giancotti, 1989; Albelda *et al.*, 1990). It has been shown that small RGD peptides, that can block by competition tumour cell integrin receptors for extracellular matrix (Hynes, 1987), are able to reduce metastasis formation *in vivo* (Humphries *et al.*, 1988; Saiki *et al.*, 1989).

Tumour cell adhesion to endothelial cells is also important for the metastatic process. Some authors found a positive correlation between cell adhesion to endothelial cells or to subendothelial matrix proteins and metastatic potential of tumour cell lines (Belloni & Tressler, 1989) and in particular of melanoma cells (including B16) (Chang *et al.*, 1992; Kojima *et al.*, 1992; Zhu *et al.*, 1992). In these papers, inhibition of both adhesion and metastasis was achieved by specific antibodies blocking different adhesion molecules. Here we showed that H-2K$^b$ gene transfection in H-2-negative melanoma cells leads to both a decreased adhesion to endothelial cells and matrix proteins and to a dramatic reduction in the metastatic ability.

It has been previously reported that melanoma cells adhere more efficiently to IL-1-treated endothelium (Rice & Bevilacqua, 1989; Martin-Padura *et al.*, 1991). This phenomenon was related to an increase in the number and incidence of artificial and spontaneous metastasis in IL-1-treated animals injected with melanoma cell lines (Giavazzi *et al.*, 1990). We reported here that, in contrast to the parental line and Neo clones, IL-1 treatment of the endothelium did not change the ability of H-2K$^b$ transfectants to adhere to it.

The augmentation of melanoma adhesiveness to cytokine-treated HEC has been proven to be mediated by the recognition of the endothelial adhesive membrane protein VCAM-1 by melanoma counter receptor VLA-4 (Rice & Bevilacqua, 1989; Martin-Padura *et al.*, 1991). Since $\alpha_4$ expression was indeed depressed in H-2K$^b$ transfectants, it is likely that H-2K$^b$ transfection impairs VLA-4/VCAM-1 typical recognition.

The mechanism underlying the profound depression of adhesion both to the endothelium and matrix proteins observed in H-2K$^b$ transfectants cannot however be completely explained by an inhibition of $\alpha_4\beta_1$ expression. This integrin can bind fibronectin but there is no evidence that it can interact with laminin and vitronectin. Furthermore VCAM-1 is expressed on resting endothelium only in minimal amounts (Rice *et al.*, 1990). This suggests that also other mechanisms play a role in H-2K$^b$-induced decrease of adhesive functions. Actin cytoskeletal organisation was not important since H-2K$^b$ transfectants showed a normal microfilament pattern and spreading. A possibility could be that H-2 glycoproteins sterically hinder cell adhesion molecules required for melanoma adhesion to endothelial cells and to extracellular matrix. We are currently expanding the panel of available reagents recognising murine adhesion molecules, to obtain a comprehensive evaluation of the differences between Neo and Kb cells.

In conclusion our data indicate that expression of a single class I MHC gene can significantly alter tumour cell adhesive interactions. This alteration, along with the reported interference with the immune system of the hosts, could contribute to determine the decreased metastatic ability.

This study was supported by grants from Associazione Italiana per la Ricerca sul Cancro, Milano, from Ministero dell'Università e della Ricerca Scientifica e Tecnologica, Roma, and from the National Research Council, Special Project 'Applicazioni Cliniche della Ricerca Oncologica', Italy. L.L. is in receipt of a Ph.D. fellowship from Ministero dell'Università e della Ricerca Scientifica e Tecnologica, Italy.

### References

ALBEDA, S.M., METTE, S.A., ELDER, D.E., STEWART, R.M., DAMJANOVICH, L., HERLYN, M. & BUCK, C. (1990). Integrin distribution in malignant melanoma: association of the beta3 subunit with tumor progression. *Cancer Res.*, **50**, 6757–6764.

AUERBACH, R., LU, W., PARDON, E., GUMOWSKI, F., KAMINSKI, G. & KAMINSKI, M. (1987). Specificity of adhesion between murine tumor cells and capillary endothelium: an *in vitro* correlate of preferential metastasis *in vivo*. *Cancer Res.*, **47**, 1492–1496.

BALCONI, G. & DEJANA, E. (1986). Cultivation of endothelial cell. Limitation and perspective. *Med. Biol.*, **64**, 231–245.

BARTLETT, P.F. & EDIDIN, M. (1978). Effect of the H-2 gene complex rates of fibroblast intercellular adhesion. *J. Cell. Biol.*, **77**, 377–388.

BELLONI, P.N. & TRESSLER, R.J. (1989). Microvascular endothelial cell heterogeneity: interactions with leukocytes and tumor cells. *Cancer Metast. Rev.*, **8**, 353–389.

CARLOS, T.M., SCHWARTZ, B.R., KOVACH, N.L., YEE, E., ROSA, M., OSBORN, L., CHIROSSO, G., NEWMAN, B., LOBB, R. & HARLAN, J.M. (1990). Vascular cell adhesion molecule-1 mediates lymphocyte adherence to cytokine activated cultured human endothelial cells. *Blood*, **76**, 965–970.

CHANG, Y.S., CHEN, Y.Q., TIMAR, J., NELSON, K.K., GROSSI, I.M., FITZGERALD, L.A., DIGLIO, C.A. & HONN, K.V. (1992). Increased expression of alphaIIbbeta3 integrin in subpopulations of murine melanoma cells with high lung-colonizing ability. *Int. J. Cancer*, **51**, 445–451.

CONFORTI, G., ZANETTI, A., COLELLA, S., ABBADINI, M., MARCHISIO, P.C., PYTELA, R., GIANCOTTI, F.G., TARONE, G., LANGUINO, L.R. & DEJANA, E. (1989). Interaction of fibronectin with cultured humans endothelial cells. Characterisation of the specific receptor. *Blood*, **73**, 1576–1582.

CURTIS, A.S.G. & ROONEY, P. (1979). H-2 restriction of contact inhibition of epithelial cells. *Nature*, **281**, 222–223.

DE GIOVANNI, C., PALMIERI, G., NICOLETTI, G., LANDUZZI, L., SCOTLANDI, K., BONTADINI, A., TAZZARI, P.-L., SENSI, M., SANTONI, A., NANNI, P. & LOLLINI, P.-L. (1991). Immunological and nonimmunological influence of H-2Kb gene transfection on the metastatic ability of B16 melanoma cells. *Int. J. Cancer*, **48**, 270–276.

DEJANA, E., COLELLA, S., LANGUINO, L.R., BALCONI, G., CORBASCIO, G.C. & MARCHISIO, P.C. (1987). Fibrinogen induces adhesion, spreading and microfilament organization of human endothelial cells *in vitro*. *J. Cell. Biol.*, **104**, 1403–1411.

DEJANA, E., BERTOCCHI, F., BORTOLAMI, M.C., REGONESI, A., TONTA, A., BREVIARIO, F. & GIAVAZZI, R. (1988). Interleukin-1 promotes tumor cell adhesion to cultured human endothelial cells. *J. Clin. Invest.*, **82**, 1466–1470.

DEJANA, E., LAMPUGNANI, M.G., GIORGI, M., GABOLI, M., FEDERICI, A.B., RUGGERI, Z.M. & MARCHISIO, P.C. (1989). Von Willebrand factor promotes endothelial cell adhesion via an Arg-Gly-Asp-dependent mechanism. *J. Cell. Biol.*, **109**, 367–375.

DOHERTY, P.C., KNOWLES, B.B. & WETTSTEIN, P.J. (1984). Immunological surveillance of tumors in the context of major histocompatibility complex restriction of T cell function. *Adv. Cancer Res.*, **42**, 1–65.

ELLIOTT, B.E., CARLOW, D.C., RODRICKS, A.-M. & WADE, A. (1989). Perspectives on the role of MHC antigens in normal and malignant cells. *Adv. Cancer Res.*, **53**, 181–245.

GATTONI-CELLI, S., STRAUSS, R.M., WILLETT, C.G., POZZATTI, R. & ISSELBACHER, K.J. (1989). Modulation of the transformed and neoplastic phenotype of rat fibroblasts by MHC-I gene expression. *Cancer Res.*, **49**, 3392–3395.

GIAVAZZI, R., GAROFALO, A., BANI, M.R., ABBATE, M., GHEZZI, P., BORASCHI, D., MANTOVANI, A. & DEJANA, E. (1990). Interleukin 1-induced augmentation of experimental metastases from a human melanoma in nude mice. *Cancer Res.*, **50**, 4771–4775.

GOPAS., J., RAGER-ZISMAN, B., BAR-ELI, M., HAEMMERLING, G.J. & SEGAL, S. (1989). The relationship between MHC antigen expression and metastasis. *Adv. Cancer Res.*, **53**, 89–115.

GORELIK, E., JAY, G., KIM, M., HEARING, V.J., DELEO, A. & MCCOY, J.P. Jr (1991). Effects of H-2Kb gene on expression of melanoma associated antigens and lectin-binding sites on B16 melanoma cells. *Cancer Res.*, **51**, 5212–5218.

GRAF, L.H., KAPLAN, P. & SILAGI, S. (1984). Efficient DNA-mediated transfer of selectable genes and unselected sequences into differentiated and undifferentiated mouse melanoma clones. *Somat. Cell Molec. Genet.*, **10**, 139–151.

HALIOTIS, T., CARLOW, D.A. & ELLIOTT, B.E. (1990). Nonimmunological aspects of MHC function in the regulation of cell proliferation and the malignant phenotype. *Cancer Cells*, **2**, 86-90.

HONDA, H. & ROSTAMI, A. (1989). Expression of major histocompatibility complex class I antigens in rat muscle cultures: the possible developmental role in myogenesis. *Proc. Natl Acad. Sci. USA*, **86**, 7007-7011.

HUMPHRIES, M.J., YAMADA, K.M. & OLDEN, K. (1988). Investigation of the biological effects of anti-cell adhesive synthetic peptides that inhibit experimental metastasis of B16-F10 murine melanoma cells. *J. Clin. Invest.*, **81**, 782-790.

HYNES, R.O. (1987). Integrins: a family of cell surface receptors. *Cell*, **48**, 549-554.

KOJIMA, N., SHIOTA, M., SADAHIRA, Y., HANDA, K. & HAKOMORI, S. (1992). Cell adhesion in a dynamic flow system as compared to static system. Glycosphingolipid interaction in the dynamic system predominates over lectin- or integrin-based mechanisms in adhesion of B16 melanoma cells to non-activated endothelial cells. *J. Biol. Chem.*, **267**, 17264-17270.

KORACH, S., POUPON,. M.F., DUVILLARD, J.A. & BECKER, M. (1986). Differential adhesiveness of rhabdomyosarcoma-derived cloned metastatic cell lines to vascular endothelial monolayers. *Cancer Res.*, **46**, 3624-3629.

LAMPUGNANI, M.G., RESNATI, M., DEJANA, E. & MARCHISIO, P.C. (1991). The role of integrins in maintenance of endothelial monolayer integrity. *J. Cell. Biol.*, **112**, 479-490.

LAURI, D., BERTOMEU, M.C., ORR, F.W., BASTIDA, E., SAUDER, D. & BUCHANAN, M.R. (1990). Interleukin-1 increases tumor cell adhesion to endothelial cells through an RGD dependent mechanism: *in vitro* and *in vivo* studies. *Clin. Expl. Metastasis*, **8**, 27-32.

LJUNGGREN, H.-G. & KARRE, K. (1990). In search of the 'missing self': MHC molecules and NK recognition. *Immunol. Today*, **11**, 237-244.

MARTIN-PADURA, I., MORTARINI, R., LAURI, D., BERNASCONI, S., SANCHEZ-MADRID, F., PARMIANI, G., MANTOVANI, A., ANICHINI, A. & DEJANA, E. (1991). Heterogeneity in human melanoma cell adhesion to cytokine activated endothelial cells correlates with VLA4 expression. *Cancer Res.*, **51**, 2239-2241.

MCCARTHY, J.B., BASARA, M.L., PALM, S.L., SAS, D.F. & FURCHT, L.T. (1985). The role of cell adhesion proteins laminin and fibronectin in the movement of malignant and metastatic cells. *Cancer Metastasis Rev.*, **4**, 125-152.

NICOLSON, G.L. (1982). Metastatic tumor cell attachment and invasion assay utilizing vascular endothelial cell monolayers. *J. Histochem. Cytochem.*, **30**, 214-220.

NICOLSON, G.L. (1988). Organ specificity of tumor metastasis: role of preferential adhesion, invasion, and growth of malignant cells at specific secondary sites. *Cancer Metastasis Rev.*, **7**, 143-188.

RICE, G.E., GIMBRONE, M.A. Jr & BEVILACQUA, M.P. (1989). Tumor cell-endothelial cell interactions. Increased adhesioin of human melanoma cells to activated vascular endothelium. *Am. J. Pathol.*, **133**, 204-210.

RICE, G.E., MUNRO, M.J. & BEVILACQUA, M.P. (1990). Inducible cell adhesion molecule 110 (INCAM-110) is an endothelial receptor for lymphocytes: a CD11/CD18 independent adhesion mechanism. *J. Exp. Med.*, **171**, 1369-1371.

RICE, G.R. & BEVILACQUA, M.P. (1989). An inducible cell surface glycoprotein mediates melanoma adhesion. *Science*, **246**, 1303.

RUOSLAHTI, E. & GIANCOTTI, F.G. (1989). Integrins and tumor cell dissemination. *Cancer Cells*, **1**, 119-126.

SAIKI, I., IDA, J., MURATA, J., OGAWA, R., NISHI, N., SUGIMURA, K., TOKURA, S. & AZUMA, I. (1989). Inhibition of the metastasis of murine malignant melanoma by synthetic polymeric peptides containing core sequences of cell adhesive molecules. *Cancer Res.*, **49**, 3815-3822.

SCHIRRMACHER, V., CHEINSONG-POPOV, R. & ARNHEITER, H. (1980). Hepatocyte-tumor cell interaction *in vitro*. I. Conditions for rosette formation and inhibition by anti-H2 antibody. *J. Exp. Med.*, **151**, 984-989.

SUNDAY, M.E., ISSELBACHER, K.J., GATTONI-CELLI, S. & WILLETT, C.G. (1989). Altered growth of a human neuroendocrine carcinoma line after transfection of a major histocompatibility complex class I gene. *Proc. Natl Acad. Sci. USA*, **86**, 4700-4704.

TANAKA, K., YOSHIOKA, T., BIEBERICH, C. & JAY, G. (1988). Role of the major histocompatibility complex class I antigens in tumor growth and metastasis. *Ann. Rev. Immunol.*, **6**, 359-380.

VARANI, J., GRIMSTAD, I.A., KNIBBS, R.N., HOVIG, T. & MCCOY, J.P. (1985). Attachment, spreading, and growth *in vitro* of highly malignant and low malignant murine fibrosarcoma cells. *Clin. Expl. Metastasis*, **3**, 45-59.

WALLICH, R., BULBUC, N., HAEMMERLING, G.J., KATZAV, S., SEGAL, S. & FELDMAN, M. (1985). Abrogation of metastatic properties of tumour cells by de novo expression of H-2K antigens following H-2 gene transfection. *Nature*, **315**, 301.

WELLICOME, S.M., THORNHILL, M.H., PITRALIS, C., THOMAS, D.S., LANCHBURY, J.S.S., PANAYI, G.S. & HASKARD, D.Q. (1990). A monoclonal antibody that detects a novel antigen on endothelial cells that is induced by tumor necrosis factor, IL-1 or polysaccharide. *J. Immunol.*, **144**, 2558-2563.

WEXLER, H. (1966). Accurate identification of experimental pulmonary metastases. *J. Natl. Cancer Inst.*, **36**, 641-645.

WILLIAMS, R.L., COURTNEIDGE, S.A. & WAGNER, E.F. (1988). Embryonic lethalities and endothelial tumors in chimeric mice expressing polyoma virus middle T oncogene. *Cell*, **52**, 121-131.

WILLIAMS, R.L., RISAU, W., ZERWES, H.G., DREXLER, H., AGUZZI, A. & WAGNER, E.F. (1989). Endothelioma cells expressing the polyoma middle T oncogene induce hemangiomas by host cell recruitment. *Cell*, **57**, 1053-1063.

ZHU, D., CHENG. C.F. & PAULI, B.U. (1992). Blocking of lung endothelial cell adhesion molecule-1 (Lu-ECAM-1) inhibits murine melanoma lung metastasis. *J. Clin. Invest.*, **89**, 1718-1724.